# A Novel Desloratadine-Benzoic Acid Co-Amorphous Solid: Preparation, Characterization, and Stability Evaluation

**DOI:** 10.3390/pharmaceutics10030085

**Published:** 2018-07-06

**Authors:** Ahmad Ainurofiq, Rachmat Mauludin, Diky Mudhakir, Sundani Nurono Soewandhi

**Affiliations:** 1 School of Pharmacy, Institut Teknologi Bandung, Ganesha 10, Bandung 40132, Indonesia; rachmat@fa.itb.ac.id (R.M.); mudhakir@fa.itb.ac.id (D.M.); 2 Department of Pharmacy, Sebelas Maret University, Ir. Sutami 36A, Surakarta 57126, Indonesia

**Keywords:** desloratadine, benzoic acid, co-amorphous, solubility, stability, melt-quenching

## Abstract

Low physical stability is the limitation of the widespread use of amorphous drugs. The co-amorphous drug system is a new and emerging method for preparing a stable amorphous form. Co-amorphous is a single-phase amorphous multicomponent system consisting of two or more small molecules that are a combination of drugs or drugs and excipients. The co-amorphous system that uses benzoic acid (BA) as an excipient was studied to improve the physical stability, dissolution, and solubility of desloratadine (DES). In this study, the co-amorphous formation of DES and BA (DES–BA) was prepared by melt-quenching method and characterized by differential scanning calorimetry (DSC), Fourier transform infrared spectroscopy (FTIR), powder X-ray diffraction (PXRD), and polarized light microscopy (PLM). Dissolution, solubility, and physical stability profiles of DES–BA were determined. The DES crystals were converted into DES–BA co-amorphous form to reveal the molecular interactions between DES and BA. Solid-state analysis proved that the co-amorphous DES–BA system (1:1) is amorphous and homogeneous. The DSC experiment showed that the glass transition temperature (Tg) of tested DES–BA co-amorphous had a higher single Tg compared to the amorphous DES. FTIR revealed strong interactions, especially salt formation. The dissolution rate and solubility of co-amorphous DES–BA (1:1) obtained were larger than the DES in crystalline form. The PXRD technique was used to assess physical stability for three months at 40 °C with 75% RH. The DES–BA co-amorphous system demonstrated better physical stability than a single form of amorphous DES. Co-amorphous DES–BA has demonstrated the potential for improving solid-state stability, as the formation of DES–BA co-amorphous salt increased solubility and dissolution when compared to pure crystalline DES. This study also demonstrated the possibility for developing a DES–BA co-amorphous system toward oral formulations to improve DES solubility and bioavailability.

## 1. Introduction

A crystal engineering strategy was applied to modify the physicochemical properties of drugs through the formation of salts, polymorphs, solvates, hydrates, amorphous and newer forms, co-crystal and co-amorphous systems [1,2]. In recent years, amorphous drugs have become interesting subjects because of their favorable properties, such as higher solubility, faster dissolution rate, and greater bioavailability when compared to their crystalline form. This is because the amorphous compound is in the highest internal energy state of the solid material and has a higher molecular motion than the crystal compound [3,4]. However, pure amorphous drugs often show rapid recrystallization kinetics to low energy crystal states (low solubility) [5,6]. Thus, the amorphous material is thermodynamically unstable with a relatively lower physical and chemical stability than crystals [7]. Consequently, during manufacturing, processing, or storing, the amorphous material may return to the crystal form and lose its superior properties. Therefore, it is necessary to stabilize the amorphous form to take advantage of the benefits provided by drugs in an irregular state.

Many amorphous drugs are formulated to form solid dispersions with a hydrophilic polymer matrix to improve the physical stability of amorphous drugs under storage condition and dissolution [8]. Complexes formed with polymers can prevent drug crystallization by reducing the mobility of drug molecules. The molecules are kinetically trapped between the polymer chains in a high-energy non-crystalline state that leads to increased stability by increasing the value of glass transition temperature (Tg) compared with pure amorphous drugs [8,9,10]. Generally, a weak interaction is not sufficient to stabilize amorphous drugs [11]. Predictions and designs that lead to the interaction between drugs and polymers are still difficult to achieve. Despite intensive research, however, only some amorphous solid dispersion-based formulations have reached the global market [12]. Several challenges are associated with this polymer, as the dispersion of solid polymers tends to be hygroscopic. The absorbed moisture causes a decrease in glass transition temperature (Tg), which can support phase separation and facilitate the recrystallization of combined amorphous drugs [13]. In addition, in some cases, a large amount of polymers is required for solid dispersion face difficulty in manufacturing and processing [14]. Dispersion with polymers often produces poor solubility of the drug in the polymer. Limitations also exist concerning the miscibility of drugs and bad polymers, which can lead to high drug-to-polymer ratios and very large volume of the final dose, thus limiting their use, especially for high-dose drugs [9]. Furthermore, challenges during the pulverization process regarding processing into forms and dispersion scale of solid polymers have been reported [15]. Another weakness of the polymer system is poor compression [16]. This challenge typically negates the advantages of solubility and dissolution of the amorphous system, sometimes making the product unsafe and ineffective, as seen by the withdrawal of many solid dispersion products by the FDA [17]. Thus, another alternative way to overcome the physical stability of amorphous drugs is required.

The co-amorphous system is a novel discovery and one of the promising formulation approaches, as it overcomes the limitations associated with dispersion of a polymer amorphous solid. A binary amorphous system, known as co-amorphous, combines drugs with small molecules instead of polymers to form a homogeneous amorphous phase at the molecular level [18,19]. Drugs in the form of co-amorphous are amorphous complexes with special interactions, which become synthons between drugs and excipients or other drugs that possess both features of solid dispersion and co-crystals [18,20]. Chieng et al. (2009) first used the term co-amorphous, defined as a “single-phase multicomponent solid amorphous system which has no lattice periodicity and is attributed to weak and discrete intermolecular interactions between components” [20,21]. They have short-term interactions, such as hydrogen bonds of carboxylic acids, alcohols, and carboxamides, similar to the amorphous system of a single component system. However, this system differs from the co-crystal, salt or eutectic, especially its amorphous properties characterized by the existence of a broad hump (‘amorphous halo’) in X-ray diffraction [22]. Stabilization of amorphous drugs is achieved by increasing the Tg of the mixture through salt formation or intermolecular interactions. There is evidence to indicate that the co-amorphous system of two small molecule drugs increases solubility and stability for amorphous drugs through strong intermolecular interactions between two drugs [20,23]. In addition to drug combinations with pharmacologically suitable drugs, co-amorphous systems of drugs and small molecule excipients seem to be a more common concept. Other small molecules, such as sugars, amino acids, nicotinamides, urea, carboxylic acids, and saccharin, have also been explored as co-amorphous excipients, which have significantly increased solubility, dissolution rate, and physical stability of amorphous drug forms [24,25,26]. Since a small molecule excipient is a molar stoichiometric unit, the required amount of a counter molecule used as a drug additive is generally smaller than that of a polymer in solid dispersion; thus, co-amorphous can provide a smaller volume and a relatively lower cost. Compared with co-crystals, co-amorphous does not require crystallization and provides increased solubility in the amorphous state.

Importantly, the method of making co-amorphous will affect certain properties of amorphous drugs, such as glass transition temperature (Tg), dissolution, and stability [27]. Co-amorphous formulation preparation techniques can be roughly divided into fusion or melting techniques (e.g., melt-quenching and hot melt extrusion), solvent techniques (e.g., solvent casting, freeze-drying, and spray-drying), and techniques involving mechanical activation (e.g., cryogenic grinding, and pulverization) [28].

Desloratadine (DES), which has a practical property of being poorly water-soluble [29], has a proven safe and effective non-sedative antihistamine activity useful for allergic rhinitis, allergic asthma, and urticaria [30]. Several studies have attempted to improve DES solubility. Previous studies to improve the solubility of DES include the formation of complex inclusion of DES with β-cyclodextrin in a solution [31], and DES made in the form of hemifumarate salts to increase solubility [32]. A solid dispersion study of DES with povidone and crospovidone showed that it could increase the rate of drug dissolution [33]. Another study to increase the dissolution rate of DES with solid dispersion using poloxamer obtained results in amorphous form, which play a role in increasing intrinsic solubility [34]. However, there are some disadvantages of solid dispersion techniques, such as hygroscopy, difficulty of manufacturing, limited miscibility of drug-polymer, phase separation, and poor compression of polymers [19]. Therefore, other studies have attempted to make DES stable as well as improve its solubility and dissolution by making multicomponent crystal salt [35,36]. Notably, there has been no research effort to produce a DES system in a co-amorphous form.

In this study, the co-amorphous form of DES with benzoic acid (DES–BA), a pharmaceutically acceptable co-former, was prepared to improve its physicochemical properties, that is, dissolution rate, solubility, and stability of DES made by a melt-quenching technique. The physicochemical properties of co-amorphous DES–BA were characterized by X-ray powder diffraction (XRPD), differential scanning calorimetric (DSC), and Fourier transform infrared spectroscopy (FTIR) to verify changes in their solid-state. Solubility, dissolution, and physical stability were also investigated.

## 2. Materials and Methods

### 2.1. Materials

Desloratadine (pharmaceutical grade), which is a polymorphic form I, was purchased from Xi’an Wango Biopharm Co., Ltd. (Shaanxi, China). BA (analytical grade) and hydrochloric acid (analytical grade) were obtained from Merck (Darmstadt, Germany), respectively. Methanol (analytical grade) was obtained from J. T. Baker Inc. (Phillipsburg, NJ, USA). 

### 2.2. Methods

#### 2.2.1. Preparation of DES with BA Physical Mixture

To obtain a binary mixture system homogeneously, an equimolar composition of 1:1 DES and BA mixture was prepared by mixing gently in the mortar for five minutes. The physical mixture of DES and BA obtained was analyzed immediately with PXRD, DSC, and FTIR after preparation to avoid a moisture effect.

#### 2.2.2. Preparation of Amorphous DES and Co-Amorphous DES–BA

Single amorphous DES and the co-amorphous DES–BA system were prepared using the melt-quenching method. Briefly, DES crystals were melted in containers at 158 °C. Melting time of samples was not more than two minutes, and they were then chilled in a freezer to form amorphous DES. Meanwhile, in the co-amorphous DES–BA with equimolar composition of 1:1 mixture of DES and BA, DES was melted first because its melting point is higher than BA. Afterwards, BA was added to the cooled melted DES and heated together until everything was melted and then stirred until homogeneous. The result was directly chilled in the freezer. The amorphous DES and co-amorphous DES–BA were sieved through 100 mesh (150 μm) and stored in a vacuum desiccator above anhydrous calcium chloride at 4 °C for further characterization.

#### 2.2.3. Preparation of Desloratadine Polymorph Form II

DES (500 mg) was dissolved into a methanol solvent until the consistency of the solution was clear. The rapid methanol evaporation process was carried out by heating at 70 °C until dry crystals of DES were obtained. The result obtained was DES in polymorphic form II.

#### 2.2.4. Preparation of Desloratadine-Benzoic Acid Salt

The equimolar mixture of DES and BA was dissolved in methanol at 35 °C until a clear solution was obtained. The resulting solution was then evaporated using a Buchi Rotavapor R-215 (Flawil, Switzerland) at 50 °C, followed by a Buchi Heating Bath B-491 (Flawil, Switzerland) and sucked at a pressure of 208 mbar using the Buchi V-850 vacuum controller. The powder obtained from the multicomponent crystal of DES–BA salt was collected and stored at room temperature for further analysis.

#### 2.2.5. Solid-State Characterization of Co-Amorphous Desloratadine-Benzoic Acid

##### Powder X-ray Diffraction

The PXRD measurements were performed using Bruker D8 Advance X-Ray Diffractometer (Madison, WI, USA). All scanning was performed at 2°/min level and diffraction angle of 2θ from 5–45° using a resolution of 0.02°. Samples were characterized using Cu-kα radiation (1.5406 Ǻ wavelength) at 40 kV with a 35 mA current.

##### Differential Scanning Calorimetry

DSC characterization was performed using LINSEIS PT-1600 (Robbinsville, NJ, USA), which was calibrated for cell and temperature constants using indium. The following parameters were used: temperature at 25–300 °C under nitrogen cleansing of 100 mL/min, a heating rate of 10 °C/min, Al_2_O_3_ container, 2–5 mg sample placed into a pot, and an empty pot used as a reference.

##### Fourier Transform Infrared Spectroscopy

The spectrum samples of Fourier transform infrared (FTIR) were performed using IR Prestige-21 Shimadzu (Kyoto, Japan) and analyzed within a wave range of 400–4000 cm^−1^ with resolution of 2 cm^−1^. Afterwards, the peak of the obtained spectrum was compared. The intensity and shift of the vibration peak was observed.

##### Polarized Light Microscopy

Co-amorphous DES–BA and amorphous DES were observed separately. Observations were made after storage at 0, 3, 8, 17, 30, 45, and 90 days for co-amorphous from DES–BA, and at 0, 2, 7, 15, and 30 days for amorphous DES. Samples were placed on top of a glass object and dripped with liquid paraffin to clarify the observations. Sample habits were observed using polarization of the BX-50 microscope (Olympus, Tokyo, Japan). Images were collected using the Olympus SC-30 digital camera (Tokyo, Japan) and analyzed using GetIT AnalySIS software (Tokyo, Japan).

#### 2.2.6. Physical Stability

In the stability study, the co-amorphous DES–BA system (1:1) and amorphous DES were placed into glass bottles and stored in a Hotpack 317332 Climatic Chamber (Philadelphia, PA, USA) at 40 °C and 75% relative humidity. Co-amorphous DES–BA samples were analyzed repeatedly with XRPD at 0, 3, 8, 17, 30, 45, and 90 days, whereas the amorphous DES was analyzed with XRPD at 0, 2, 7, 15, and 30 days to investigate the stability of the material.

#### 2.2.7. Solubility

The solubility test was performed under different media, that is, water and 0.1 N HCl. An excess amount (2 g) of samples was added, to a test tube containing 10 mL of medium to saturate. The reaction tube was shaken in an orbital shaker (120 Hz, 37 ± 0.5 °C) for 24 h until an equilibrium condition was reached. The solution was filtered using a 0.22-μm nylon filter, and the solution was then analyzed using a Beckman Coulter DU720 spectrophotometer (Brea, CA, USA) at 290 nm wavelength using a validated analysis method.

#### 2.2.8. Dissolution

The dissolution experiments for DES and DES–BA co-amorphous were performed using 900 mL water and 0.1 N HCl as the dissolution medium. The dissolution tool of Hanson SR8Plus (Hanson Research, Chatsworth, CA, USA) according to USP apparatus II (paddle method) was used at 37 ± 0.5 °C, and the solution was stirred at 50 rpm. Samples equivalent to 25 mg DES were added, and 5 mL aliquots were drawn at different intervals (i.e., 5, 10, 15, 20, 30, 45, and 60 min), filtered using a 0.45-μm membrane filter, and then replaced with 5 mL of new dissolution medium. Each experiment was performed three times, and the results of the trials were averaged. The results were analyzed using the Beckman Coulter DU^®^720 General Purpose Spectrophotometer (Brea, CA, USA) at a validated wavelength of 290 nm.

## 3. Results and Discussion

### 3.1. Making of Amorphous DES and Co-Amorphous DES–BA

Many lab-scale methods have been reported for the manufacturing of amorphous systems. In this study, the thermal method (melt-quenching) was chosen with the consideration that DES materials used for production require only small quantities and drugs including thermostable to the heat. In addition, some studies have compared methods such as ball-milled, cryo-milled, spray-dried, and melt-quenching on indomethacin manufacturing, specifically, cimetidine and naproxen as well as co-amorphous cimetidine [37]. The amorphous form of naproxen made by ball milled, cryo-milled, and spray-dried methods was unstable and recrystallized rapidly compared to melt-quenching. Another study stated that melt-quenched simvastatin was more stable than cryo-milled simvastatin [27,38]. However, grinding methods, such as ball-milled and cryo-milled, are the most widely used methods due to their ease of handling.

In the making of co-amorphous formulations, small molecules as co-formers are the first step to consider because they are influential on the stability of the manufacturing and can improve the physicochemical properties of a drug [39]. In this study, benzoic acid (BA) was selected because previous studies have proven it to be a well-formed multicomponent of DES crystals by improving the physicochemical properties of drugs that were not formed with other co-formers [35]. Meanwhile, the effort of DES co-amorphous formation has been prepared with several small molecules besides BA, e.g., succinic acid, fumaric acid, citric acid, malonic acid, maleic acid, oxalic acid, nicotinamide, and nipagin. However, most of the results are less satisfactory; DES with citric acid is amorphous but very hygroscopic, which causes a sticky consistency. In fact, DES is not amorphous with most of the small molecules always obtained by oily liquid with succinic acid, fumaric acid, malonic acid, maleic acid, oxalic acid, nicotinamide, and nipagin.

For most co-amorphous systems, increased physical stability is associated with different intermolecular interactions between mixed compounds. This suggests that the molecular ratio present in the co-amorphous phase plays a more important role in terms of the physical stability of this system than the glass transition temperature (Tg). Indeed, in most studies, the best physical stability within each equimolar system is due to intermolecular interactions between components [14], which are perfect and leave nothing behind. With other molar ratios, excess components will crystallize first and relatively quickly, unless mechanisms other than strong molecular interaction stabilize the mixture [40]. Therefore, this study directly made co-amorphous DES–BA with a 1:1 molar ratio.

### 3.2. Characterization of Co-Amorphous DES–BA

#### 3.2.1. X-ray Powder Diffraction Study

**Figure 1:**
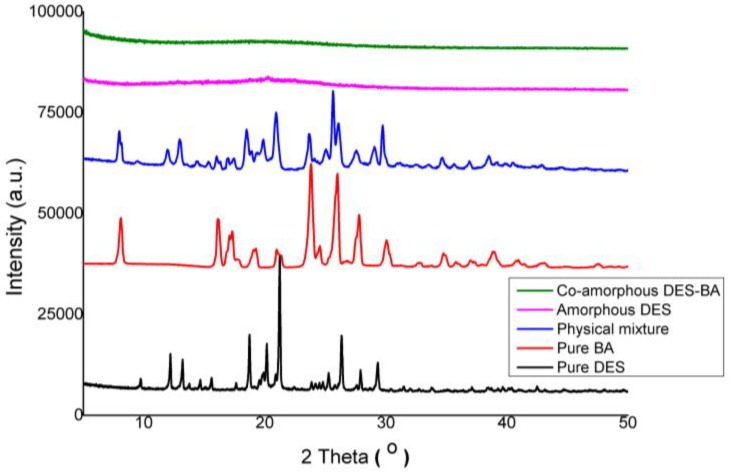
PXRD patterns of pure desloratadine (DES) (black line), pure benzoic acid (BA) (red line), physical mixture (blue line), pure amorphous DES (pink line), and co-amorphous DES–BA (green line).

X-ray diffraction from DES and BA individually, the DES–BA (1:1) physical mixture, amorphized DES, and DES–BA (1:1) co-amorphous formation are presented in Figure 1. The sharp peaks of individual powders of DES and BA as well as physical mixtures of DES–BA, which showed the combined peaks of DES and BA, clearly indicate the shape of crystals. The physical mixture has a clear overlapping diffraction peak, which is the superposition of the two components. In contrast, the pure amorphous DES powder samples and a DES–BA mixture synthesized to co-amorphous form with the melt-quenching method resulted in a distinctive halo pattern with no diffraction peak showing the conversion of the crystalline form to an amorphous one. The loss of the crystal peak on XRPD can also be interpreted as the binary mixture of DES–BA prepared by the melt-quenching method being perfectly converted to a homogeneous co-amorphous form. However, other methods used to characterize the DES–BA co-amorphous system (1:1) is required to confirm co-amorphous occurrence.

#### 3.2.2. Differential Scanning Calorimetry

**Figure 2:**
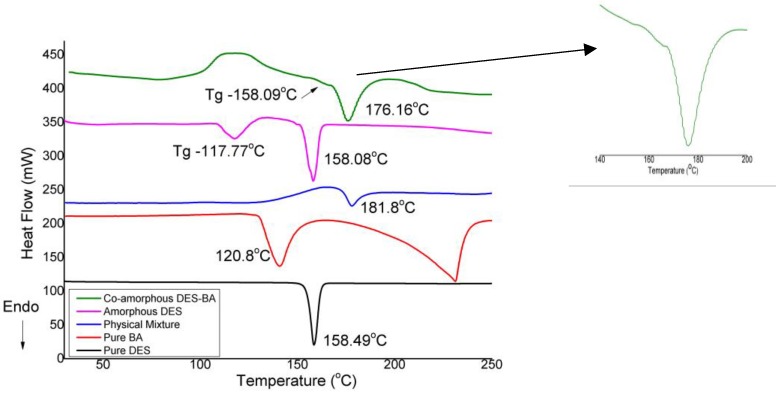
DSC thermograms of pure desloratadine (DES) (black line), pure benzoic acid (BA) (red line), physical mixture (blue line), pure amorphous DES (pink line), and co-amorphous DES–BA (green line).

DSC thermogram from DES crystals, BA crystals, physical mixture of DES–BA, amorphous DES, and the DES–BA co-amorphous system are shown in Figure 2. Sharp endothermic peaks of DES crystal and BA at 158.49 °C and 120.8 °C, respectively, reflect the melting point of the crystal material. The endothermic peak of DES and BA in the physical mixture was 181.8 °C. Changes in the melting point of the physical mixture can be caused from the mediating of the two interacting compounds by the influence of DSC temperature during the process of the apparatus, the presence of melted BA, and acting as impurities in mixed systems or eutectic formation during DSC scans at high temperature. The DSC thermogram of the DES–BA co-amorphous system indicates the absence of an endothermic peak corresponding to the melting point of the DES and BA crystals, and is clearly distinct from individual crystal components and physical mixtures, indicating the formation of a new phase. This is also proved by the XRPD pattern, which suggested the formation of a DES–BA co-amorphous system.

The DSC thermogram of amorphous DES showed endothermic peak thermal behavior at 117.77 °C as the glass transition temperature (Tg), which indicated amorphous formation. The co-amorphous DES–BA system (1:1) had a single Tg at 158.09 °C. The appearance of a single Tg for a DES–BA co-amorphous system (1:1) indicated the formation of a single homogeneous amorphous phase [41]. Therefore, the DES–BA co-amorphous system (1:1) can be perfectly mixed, as DES and BA are fully dissolved into one another during one phase. The Tg of DES–BA co-amorphous system (1:1) was 158.09 °C and higher than the amorphous DES, which was 117.77 °C. Higher Tg of the co-amorphous DES–BA system compared to amorphous DES indicates the occurrence of a strong specific interaction between DES and BA in the co-amorphous system. The DSC test result showed that the interaction between DES and BA with the melt-quenching method is present in the co-amorphous form of the DES–BA pair.

#### 3.2.3. Fourier Transform Infrared Spectroscopy

**Figure 3:**
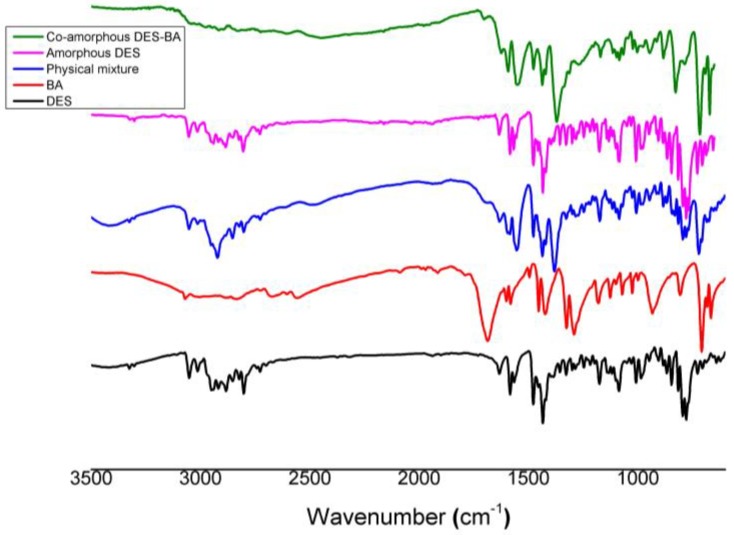
FTIR spectra of pure desloratadine (DES) (black line), pure benzoic acid (BA) (red line), physical mixture (blue line), pure amorphous DES (pink line), and co-amorphous DES–BA (green line).

FTIR was used to confirm the possibility of molecular interactions between DES and BA in co-amorphous DES–BA. The entire spectra of DES crystals, BA crystals, physical mixtures of DES–BA, amorphous DES, and co-amorphous DES–BA are presented in Figure 3 depicting differences in vibrational transitions observed in the bands in the spectrum. DES and BA each showed specific spectra; after physically mixing DES and BA, the FTIR spectrum is a combination of both components, indicating no interaction between DES and BA after a simple mixing process. Meanwhile, DES crystals with amorphous DES did not show spectral changes, which indicate no structural change when DES became amorphous. On the other hand, the FTIR spectrum of the DES–BA co-amorphous system showed a different spectral peak. In the DES–BA co-amorphous system, some characteristics of the FTIR absorption bands from 1000 to 1800 cm^−1^ for co-amorphous formation were evaluated, since major differences in the spectrum are observed in the region. Co-amorphous DES–BA was shown by the characteristic appearance of the bands of carboxylic acids at 1650–1550 cm^−1^, signifying salt formation. The formation of DES–BA co-amorphous salts was reinforced with a different FTIR pattern with DES. DES–BA co-amorphous salt molecules were characterized by stretching C=O from a group of carboxylic acids of BA at 1686.82 cm^−1^ that were not found, replaced by the band characteristics of the carboxylic acid salts at 1594.23 cm^−1^ and 1552.76 cm^−1^. In BA, the neutral carboxylic group (–COOH) had a strong carbonyl (C=O) with stretching at a peak of 1686.82 cm^−1^ and a weak carbonyl (C–O) stretching at 1291.40 cm^−1^. If deprotonation occurs during DES–BA co-amorphous salt formation, the carboxylic anion (–COO^−^) has only a single stretch (C–O) at 1379.16 cm^−1^. The formation of DES–BA co-amorphous salts showed the intermolecular interaction in the compound. Interaction can be identified by the change of vibration frequency of the functional group. The presence and shifting of specific functional groups of DES and BA as well as the formation of co-amorphous salts indicated an interaction between DES and BA, which ultimately slows the recrystallization process. The peak shift of the spectrum for the carbonyl functional group indicated its participation in ionic bonds in salt formation [42].

#### 3.2.4. Polarized Light Microscopy (PLM)

**Figure 4:**
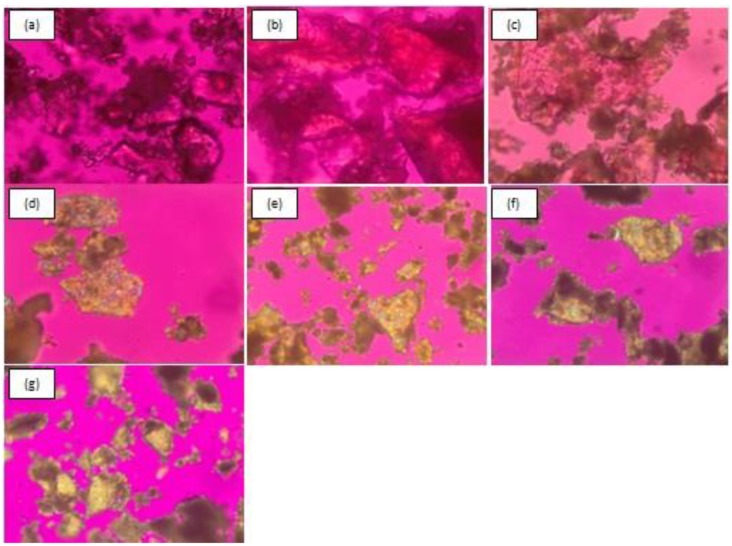
Polarized light microscopic images (magnification 400×) of crystal growth from a co-amorphous desloratadine-benzoic acid (DES–BA) system for stability studies after storing at 40 °C under 75% RH and measured after specified periods of time: fresh prepared (**a**), 3 days (**b**), 8 days (**c**), 17 days (**d**), 30 days (**e**), 45 days (**f**) and 90 days (**g**).

**Figure 5:**
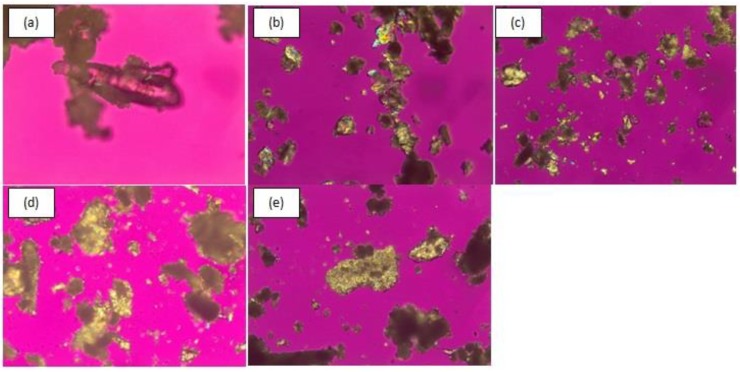
Polarized light microscopic images (magnification 400×) of crystal growth from pure amorphous desloratadine (DES) for stability studies after storing at 40 °C under 75% RH and measured after specified periods of time: fresh prepared (**a**), 2 days (**b**), 7 days (**c**), 15 days (**d**), and 30 days (**e**).

The process of recrystallization of amorphous condition can be observed after storage at 0, 3, 8, 17, 30, 45, and 90 days for co-amorphous DES–BA, and at 0, 2, 7, 15, and 30 days for amorphous DES. Observation of crystal habits was performed under a polarized microscope equipped with a digital camera. PLM is a method that can indicate the formation of new crystals. Figure 4 and Figure 5 illustrate the observations of the recrystallization of co-amorphous DES–BA and amorphous DES, respectively. Figure 4a–c do not show crystal growth; small crystals began to appear on the seventeenth day (Figure 4d). There was more growth over time, which can be observed in Figure 4e–g, and on the ninetieth day (Figure 4g), perfectly formed crystals could be seen. Meanwhile, Figure 5a shows the observation of amorphous DES only on day 0 and crystals beginning to form on the second and subsequent days (Figure 5b–e); on the fifteenth day (Figure 5d), perfect crystals had formed.

The amorphous form of a component under a polarized microscope will not appear in color, just clear so that the color matches the observation base in the microscope. The occurrence of crystals is characterized by the emergence of new colors from the base of the microscope that fit their respective crystal habit. The orientation of fragments, thicknesses, and rays absorbed or forwarded by crystal fragments influence the color variation and intensity observed. These observations were in accordance with the results of the analysis of DES–BA and amorphous DES stability using PXRD, which showed that co-amorphous DES–BA is more stable than amorphous DES.

### 3.3. Physical Stability of Co-Amorphous

**Figure 6:**
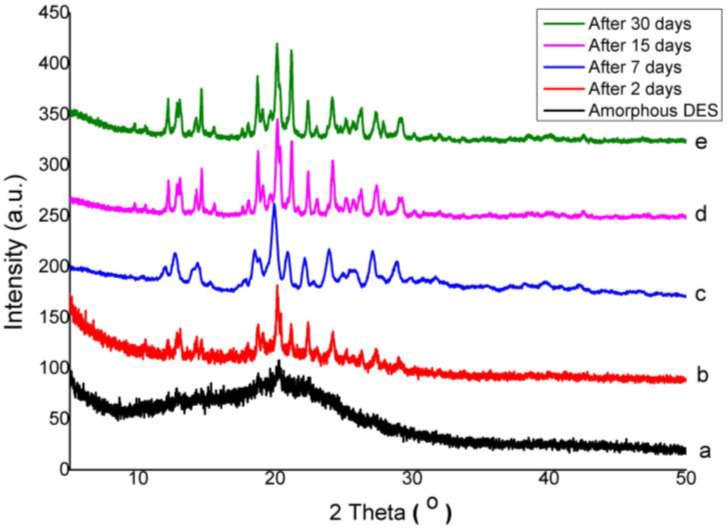
PXRD patterns of pure amorphous desloratadine (DES) for stability studies after storing at 40 °C under 75% RH and measured after specified periods of time: fresh prepared (**a**), 2 days (**b**), 7 days (**c**), 15 days (**d**), and 30 days (**e**).

**Figure 7:**
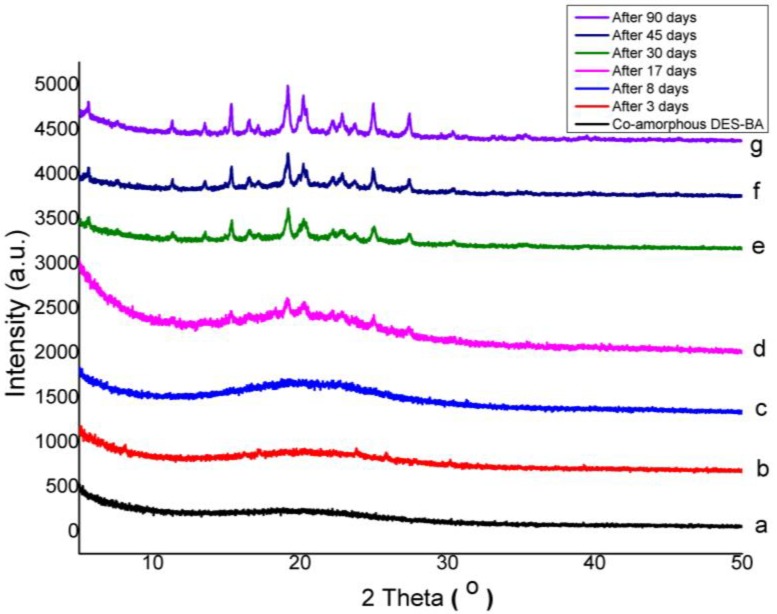
PXRD patterns of co-amorphous desloratadine-benzoic acid (DES–BA) system for stability studies after storing at 40 °C under 75% RH and measured after specified periods of time: fresh prepared (**a**), 3 days (**b**), 8 days (**c**), 17 days (**d**), 30 days (**e**), 45 days (**f**) and 90 days (**g**).

To investigate the solid-state stability of the DES–BA co-amorphous mixture and compare it with the stability of a pure amorphous DES drug, the material was stored at 40 °C, 75% RH. Physical stability of DES–BA co-amorphous system (1:1) and amorphous DES were investigated with PXRD. The PXRD patterns of amorphous DES and DES–BA co-amorphous system (1:1) before and after storage are shown in Figure 6 and Figure 7, respectively. Evaluations were performed at specific times where the test rhythm between co-amorphous DES–BA and amorphous DES was different. DES had shorter intervals at 0, 2, 7, 15, and 30 days. Meanwhile, the co-amorphous DES–BA samples were analyzed at 0, 3, 8, 17, 30, 45, and 90 days. Some small diffraction peaks of amorphous DES crystalline reflections were observed on the second day, and after seven days, the intensity of the diffraction peaks increased and changed with increasing storage time. On the 15th day, the diffraction peak had already formed completely and was unchanged after being observed for 30 days (Figure 6). These results indicated that the physical stability of amorphous DES was poor (<7 days) and it was easily recrystallized. The amorphous DES instability can be attributed to low Tg. In ambient conditions, this led to rapid molecular movement and immediate crystallization of DES [14].

**Figure 8:**
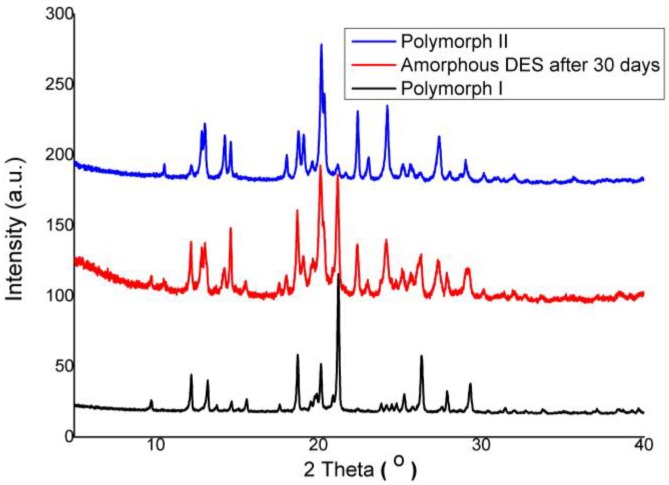
PXRD patterns of pure crystalline desloratadine (polymorph I) (black line), pure amorphous desloratadine after 30 days (red line) and pure crystalline desloratadine (polymorph II) (blue line).

However, the new crystalline DES that was formed was in contrast to the early crystalline form, which appeared to form a new polymorph after amorphization; the DES changed to a mixture of polymorph I and polymorph II form from polymorph I, which is shown in Figure 8. Meanwhile, the PXRD pattern of the DES–BA co-amorphous system (1:1), as depicted in Figure 7, showed a small diffraction peak that appeared after 17 days. Observations of the previous days showed no specific diffraction peak of the observed crystal reflection, and only the halo pattern was observed. This suggests that the physical stability of the DES–BA co-amorphous system (1:1) is better than that of amorphous DES; however, on day 30, the DES–BA co-amorphous system (1:1) had formed a large diffraction peak, which indicated full conversion to crystalline form. The DES–BA co-amorphous system (1:1) exhibited better physical stability. This stability factor may be due to the higher Tg of the DES–BA co-amorphous system compared to amorphous DES. There is a certain correlation between Tg and the physical stability of the amorphous system, in which higher Tg means higher stability. Another factor of molecular interaction is also known to play an important role in preventing recrystallization of amorphous system, resulting in a single-phase amorphous system, shown by a single Tg observed in DSC measurements. According to FTIR, it was certain that the intermolecular ionic interactions between DES and BA were present in the DES–BA co-amorphous system. The last factor, miscibility, is another prominent parameter for amorphous stabilization [43]. DES and BA were molecularly well mixed when heating with melt-quenching, which formed a homogeneous co-amorphous system in the DES–BA co-amorphous system (1:1). In a homogeneous co-amorphous system, BA acts as a recrystallization inhibitor for DES, the interaction between DES and BA inhibits the occurrence of nucleation and crystal growth and prevents the recrystallization of DES.

### 3.4. Making of DES–BA Co-Amorphous Salt

**Figure 9:**
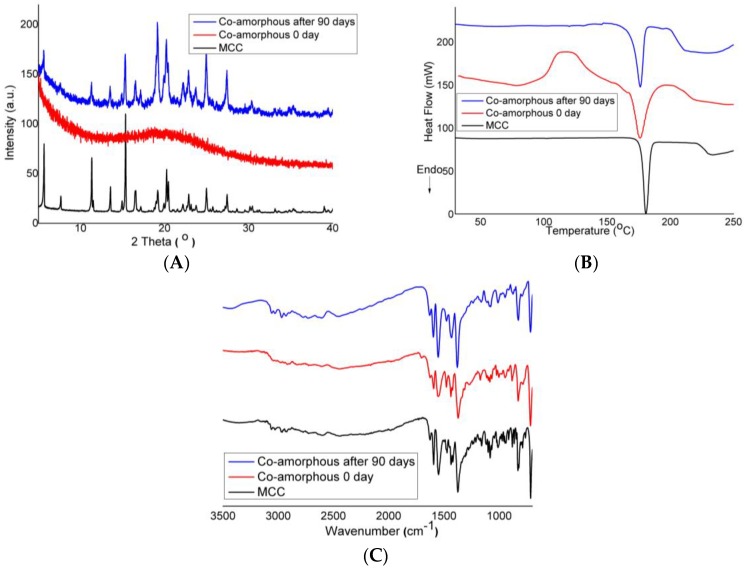
(**A**) PXRD diffractograms, (**B**) DSC thermograms and (**C**) FTIR spectra for multicomponent crystal (MCC) DES–BA (black line), co-amorphous DES–BA 0 day (red line), and co-amorphous DES–BA after 90 day (blue line).

Further evaluation was performed to understand the phenomenon of recrystallized compounds from the DES–BA co-amorphous system. Specifically, a stability study up to 90 days was conducted so that crystalline compounds could be perfectly formed during the crystallization process again. Diffractogram of the PXRD, the thermogram of DSC, and the FTIR spectrum at the end of the test as crystallized forms of the DES–BA co-amorphous system recrystallization are presented in Figure 9. Results from PXRD (Figure 9A), DSC (Figure 9B), and FTIR (Figure 9) were compared with the result from the synthesis of multicomponent crystalline salt formation from DES with BA. This has been previously investigated and published showing the similarity of the results of the compound formed as well as the recrystallization of the DES–BA co-amorphous system being DES–BA multicomponent crystal salt. The FTIR spectrum did not change when compared to the spectrum recorded on day 0; only the co-amorphous form showed the peak that occurred wider than the form of salt multicomponent crystals. This indicated that salt was formed since the manufacturing of the DES–BA co-amorphous system. Based on this data, the increased physical stability of the DES–BA co-amorphous system is proven by the strong interaction of ionic bond formation in the salt system.

### 3.5. Solubility of DES Crystal and Co-Amorphous DES–BA

The solubility of DES crystals and the DES–BA co-amorphous system after 24 h of saturation in water and in 0.1 N HCl is presented in Table 1. DES was dissolved on 0.1 N HCl and testing was performed in water and in 0.1 N HCl according to FDA rules, which also determined the effect of dissolution difference between neutral pH and acidic pH. The amorphous DES was not subjected to a solubility test due to its very low stability on the second day of crystalline formation. The unstable form of amorphous DES tends to revert to crystalline form, thus the solubility of the drug can change rapidly. Meanwhile, the results of DES crystal testing showed that it was practically insoluble in water. Compared with crystal DES, the DES–BA co-amorphous system in water and 0.1 HCl showed higher solubility. The solubility of DES–BA co-amorphous system (1:1) in water and 0.1 HCl showed an increase of approximately 27 and three folds, respectively. This significant increase in solubility indicated a significant improvement in DES solubility, which can be beneficial to dissolution and bioavailability increases in the body when DES is in the co-amorphous form. The co-amorphous formation resulted in increased solubility, which was predicted due to higher molecular mobility of the amorphous phase in the solvent medium than DES in the form of crystals. The DES molecules contained the base center of the pyridine nitrogen atom, which has a basic characteristic; thus, DES solubility was affected by the pH value. The effect of pH on solubility was proven by the solubility of DES crystalline and co-amorphous DES–BA in a 0.1 N HCl medium, which was much higher than in a water medium. These results suggested that the solubility of DES crystalline and co-amorphous DES–BA increases with decreasing pH values.

### 3.6. Dissolution of DES Crystal and Co-Amorphous DES–BA

**Figure 10:**
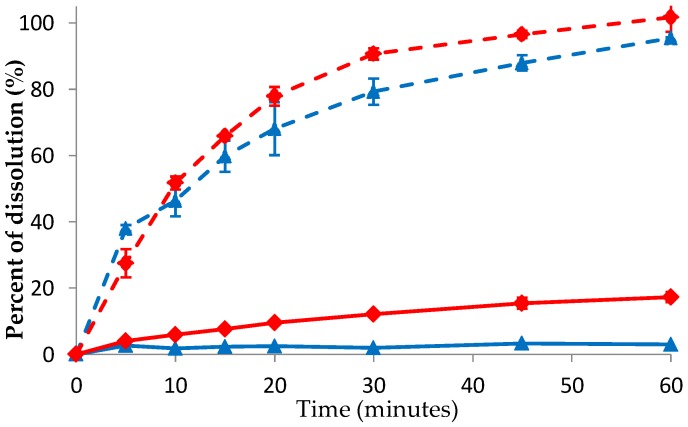
Powder dissolution profiles of pure crystalline desloratadine (DES) (blue) and co-amorphous DES–BA systems (red) in water (solid lines) and 0.1 N HCl (dashed lines) dissolution media at 37 °C.

The DES–BA co-amorphous system can increase the solubility of DES crystals, implied by its rate of dissolution. The dissolution profile observation performed on pure crystalline DES compared with DES–BA co-amorphous on 0.1 N HCl and water media is presented in Figure 10. The results showed that the formation of co-amorphous DES–BA was able to increase the dissolution rate of DES crystals. This can be seen from the dissolution efficiency (DE) value of pure DES and co-amorphous DES–BA (after 60 min) of 2.5 ± 0.6%; 11.2 ± 0.6% for water, respectively. DE results from pure DES and co-amorphous DES–BA were 70.9 ± 2.6%; 81.8 ± 0.5% for 0.1 N HCl, respectively. The DE percentage was used to compare the amount of drug released in water and 0.1 N HCl. Co-amorphous DES–BA increased the dissolution rate by approximately 4.5-fold in water and 1.2 times faster in 0.1 HCl, which showed no significant difference of drug release in 0.1 N HCl. This was based on similarity factor-*f2* and different factor-*f1*. In general, two dissolution profiles are similar if the value of *f1* < 15 and the value of *f2* > 50 [44]. The comparison of the co-amorphous DES–BA dissolution profile with pure crystalline DES in 0.1 N HCl yielded *f1* values of 12.24 and *f2* of 53.15, indicating that both profiles were similar in the medium. Meanwhile, the comparison of the co-amorphous DES–BA dissolution profile with pure crystal DES on water yielded an *f1* value of 75.75 and *f2* of 52.41, which means that the two profiles were different in the water medium. Increased co-amorphous DES–BA dissolution of DES crystals in the water medium was greater than the 0.1 N HCl medium. However, when comparing the value of DE, the 0.1 N HCl medium was higher than the water medium, which indicated that the amount of DES released in the 0.1 N HCl medium was greater. The ease of dissolution speed in the acid medium was because DES more easily ionizes in an acidic atmosphere. As expected, crystalline DES had a slow and low dissolution in the water medium compared to DES–BA in co-amorphous form. On the other hand, the amount of drug dissolved by the DES–BA co-amorphous system (1:1) at any time point is greater than that of the crystalline DES in both 0.1 HCl and water media. This suggests that the dissolution rate of the DES–BA co-amorphous system (1:1) was faster than the crystalline DES.

## 4. Conclusions

In this study, BA was successfully used as a small molecule excipient to create a co-amorphous system from DES to stabilize amorphous DES as well as to increase the solubility and dissolution of DES over its crystalline form. The DES–BA co-amorphous system was obtained through the melt-quenching technique. The solid-state analysis of PXRD, DSC, and FTIR proved that the DES–BA co-amorphous system (1:1) is a mixture of amorphous and homogeneous molecules, which had a higher single Tg from amorphous DES. Further, FTIR analysis showed strong interactions of salt formation between DES–BA (1:1). The solubility and dissolution of a co-amorphous DES–BA system (1:1) increased significantly in magnitude from DES in crystalline form. The DES–BA co-amorphous system (1:1) had better physical stability than amorphous DES after storage for three months at 40 °C with 75% RH. Physical stability of a highly unstable amorphous DES drug increased in the DES–BA co-amorphous system (1:1), which could be associated with increased Tg of the amorphous DES form as well as the formation of ionic salt bonds from DES–BA.

## Figures and Tables

**Table 1:** Solubilities data of pure crystalline desloratadine (DES) and co-amorphous DES–BA systems in water and 0.1 N HCl at 37 °C.

Compound	Solubility (Mole) in	
	Water	HCl 0.1 N
**DES**	1.03 × 10^−3^ ± 3.22 × 10^−5^	1.16 × 10^−1^ ± 2.25 × 10^−4^
**Co-amorphous DES–BA**	1.97 × 10^−2^ ± 6.01 × 10^−4^	2.55 × 10^−1^ ± 2.52 × 10^−3^
